# Design and Development of an IoT Smart Meter with Load Control for Home Energy Management Systems

**DOI:** 10.3390/s22197536

**Published:** 2022-10-05

**Authors:** Omar Munoz, Adolfo Ruelas, Pedro Rosales, Alexis Acuña, Alejandro Suastegui, Fernando Lara

**Affiliations:** Facultad de Ingeniería, Universidad Autónoma de Baja California, Blvd. Benito Juárez S/N, Mexicali 21280, Mexico

**Keywords:** smart meter, power meter, internet of things, load control, energy meter, smart socket

## Abstract

Electricity consumption is rising due to population growth, climate change, urbanization, and the increasing use of electronic devices. The trend of the Internet of Things has contributed to the creation of devices that promote the thrift and efficient use of electrical energy. Currently, most projects relating to this issue focus solely on monitoring energy consumption without providing relevant parameters or switching on/off electronic devices. Therefore, this paper presents in detail the design, construction, and validation of a smart meter with load control aimed at being part of a home energy management system. With its own electronic design, the proposal differs from others in many aspects. For example, it was developed using a simple IoT architecture with in-built WiFi technology to enable direct connection to the internet, while at the same time being big enough to be part of standardized electrical enclosures. Unlike other smart meters with load control, this one not only provides the amount of energy consumption, but rms current and voltage, active, reactive, and apparent power, reactive energy, and power factor—parameters that could be useful for future studies. In addition, this work presents evidence based on experimentation that the prototype in all its readings achieves an absolute percentage error of less than 1%. A real-life application of the device was also demonstrated in this document by measuring different appliances and switching them on/off manually and automatically using a web-deployed application.

## 1. Introduction

Energy consumption all over the world is increasing mainly because of population growth, urbanization, and new technological trends that need a large amount of electricity to work, such as smartphones, electric cars, and the mining of cryptocurrencies. However, what is the problem with the high usage of power? We can examine this question from different perspectives, such as the production approach. According to [1], fossil fuels are the main source of energy worldwide, making up 62% of total consumption by 2021. The problems with this class of resources are that they are non-renewable, so the more they are used, the faster they disappear, and they also contribute significantly to global pollution and climate change.

Additionally, many power plants or equipment installed inside the distribution infrastructure are not ready to handle the new levels of energy consumption that are required by trend technologies. This results in inadequate power supply during peak hours for end-users or even complete power outages [2]. The above issues are common in microgrids, which are decentralized power systems composed of small, diverse sources of energy that operate independently or in parallel with the main grid.

The increase in power consumption comes with a series of challenges that can be addressed through better energy efficiency, which can be encouraged by the implementation of Smart Grids (SGs). This distribution method enables a balance between supply and consumption through an effective management based on the use of modern technologies of measuring and communication [2,3,4,5].

Traditional electric grids suffer from significant limitations because they do not have the capability of anticipating or responding to sudden failures that may occur within the structure. The nature of these power grids is their monodirectional communication with end-users. Therefore, the supplier company does not receive timely feedback about problems presented that might help it resolve them [6,7]. However, the bidirectionality of smart grids, which allow both electricity production and demand to be coordinated, may result in a better energy efficiency and in a higher level of customer comfort. For example, in a conventional system when the required energy is greater than the available, the supply company chooses to carry out total load shedding in areas of lesser commercial interest, whereas in smart grids, information is delivered practically instantly from the end user, so strategic areas or even appliances can be located to reduce consumption [2].

Following the information presented, how does a power grid become smart? To answer this question, we need to take into account the instrument that enables the main feature of SGs, that is, bidirectional communication. This characteristic is possible due to the Smart Meter (SM), which is considered the key component inside this distribution architecture. The smart meter is capable of measuring many electrical parameters, displaying locally or remotely the gathered information and sometimes controlling loads [2,3,5,6].

The efficient disconnection of loads due to insufficient energy generation is one of the most important problems in the field of smart grids; however, the consumer domain has been the least explored [8]. In isolated microgrids, for example, this type of situation is common, since the electricity produced is heavily reliant on renewable resources that are available and/or stored. In this situation, if the available power is divided by the number of dwellings inside the grid, the power will be variable. Therefore, this type of context is where load control management at the appliance level plays an extremely significant role. In recent years, in order to overcome the problem of the total electrical blackout, an important area of research has been attracting growing interest since it focuses on the design of Home Energy Management Systems (HEMS) that benefits both utility companies and end-users [9].

In HEMS, the main goal is to ensure the user’s comfort while minimizing energy consumption so as to achieve a balance between the supplier and demand. In energy management on the domestic demand side, during the maximum usage window, there are multiple limitations to optimally schedule loads. According to [10], household devices can be categorized into two types: schedulable and non-schedulable. Moreover, schedulable appliances can be interruptible and non-interruptible [2]. For example, a water heater can be considered a programmable non-interruptible appliance, and a garden water pump can be a programmable interruptible appliance.

The Internet of things (IoT) is an ideal architecture for the creation of energy management systems that address the interaction with human beings, mainly because the devices with an IoT scheme are those that are in contact with an environment and provide feedback to the people through the internet [11]. Considering the above, and the importance of smart meters and the management of household appliances within HEMS in a smart grid system, this work presents the design and development of a smart meter within an IoT scheme aimed at monitoring and controlling loads on the domestic demand side according to their energy consumption, and thus consequently avoiding collapses or blackouts. The development of this device, which is called Smart Meter with Load Control (SMLC) in this document, took into account a few areas of opportunity found in related projects that are studied in the next section. As a result, our work differs from the others in the following ways:

- Simple IoT architecture (no gateway needed);
- Own design electronic implementation;
- Non-invasive electronic instrumentation;
- Integration of electrical parameters monitoring and load control;
- Scalability in standardized electrical installations;
- Presentation of calibration and validation of measurements.

The hardware application of the prototype presented in this document was carried out in different configurable priority environments, and the experiments took place in a laboratory space. However, the developed device can also be used to optimize the use of the energy generated by solar power plants, wind power plants, or some other green energy sources used in isolated systems such as in a microgrid.

The content of this paper has been organized as follows: After this introductory section, a review of the related works is presented in Section 2. The description and the process of development of the proposed device can be found in Section 3. Section 4 presents details of the experimental setup, an implementation in a real environment, the performance analysis, and results. Finally, the paper is concluded in Section 5.

## 2. Related Works

In the state of the art, different projects were found which address the management of loads at a domestic level from different approaches. For instance, Khan et al. in [12] conducted a systematic review of various home energy management schemes. Several topics were discussed, such as the advantages of HEMS, the coordination of Distributed Energy Resources (DER) (local generation) and/or appliances mixed with different tariff schemes that lead to an efficient electrical energy usage, and also the challenges of hardware that each architecture faces. In addition, Qureshi et al. investigated in [13] the existence of energy management systems for smart homes. According to the flaws that they found in the reviewed projects, they proposed an energy management scheme for smart homes based on the Internet of Things (IoT). Their design has a security mechanism to control end-to-end communication and the use of smart scheduling and time management for controllable and non-controllable household loads in order to monitor and reduce energy consumption.

Additionally, some researchers have studied the effects resulting from the demand control. For example, in [14], the National Renewable Energy Laboratory (NREL) of the United States conducted a study to identify the most effective way to reduce plug load energy use, using three different approaches. One of them and the most effective method was an automated energy management system which turns off equipment when it is unused for a certain period of time. In addition, 126 persons were tested with this technique and obtained a 21% energy reduction from the baseline.

Klein et al. in [15] simulated the operation of a multi-agent system. Their strategy is about taking real information from a building and combining it with parameters given by the occupants in order to manage and coordinate the different devices inside the building. A 12% reduction in energy consumption and a 5% improvement in occupant comfort are the impact they achieved. The proposal was never implemented in the real world. Similarly, a comprehensive automation system for buildings was discussed in [16], where they demonstrated, through a simulation, how the use of electrical energy is reduced by controlling objects like heating ventilation, air-conditioning, lighting, and plugs.

On the other hand, some other studies are focused on the development or implementation of algorithms in the demand-side energy management framework. That is the case of Ahmed et. al in [17]. For HEMS architecture, they created a new real-time load controller with a scheduling technique based on a Binary Tracking Search Algorithm (BBSA). The goal of this project is to achieve energy savings and limit household peak demand based on the scheduled operation of various appliances according to specific time, resident comfort restrictions, and priorities. Similarly, ref. [18] implemented a reinforcement learning algorithm to a home energy management system with the purpose of optimizing the household electric appliances power demand. It is important to highlight that, in the presented work, a smart meter is the source of data for the applied algorithm. According to the simulations, the approach this research took can save between 6.23% and 11.54% of electricity costs.

Rocha et al. published in [19] an artificial intelligence (AI) algorithm for energy management on the demand side in smart homes. With a new methodology, they combined three AI techniques to solve the planning of power demand in smart homes and reach a harmony between the cost of energy and user comfort. Using the techniques of Elitist Non-dominated Sorting Genetic Algorithm II, Support Vector Regression and K-means clustering, demand management was implemented taking into account the fluctuations in the price of electricity over time and the priority of appliances. Furthermore, they were able to consider forecasts of a distributed generation for the next day and determine user comfort levels.

Other authors have also covered the topic of HEMS from the perspective of developing and implementing devices for switching on/off household appliances, or measuring the amount of energy consumed by those appliances. For instance, Kang et al. introduced a light-powered remote control system that consumes absolute zero power in standby mode. The goal of this scheme is to reduce the energy usage of appliances when they are in standby mode. In their design, a 15 mW laser diode is mounted on a commercial remote controller. A 2 cm × 2.5 cm photovoltaic array, an autonomous connection circuit (ACC), and a latch type power relay are mounted on a receiver unit it does not have any power supply. This receiver is a bridge between the appliance and the AC power line, so it can completely de-energize equipment when they receive the shutdown signal from the remote control. The receiver does not have any power supply, but when it receives light, it energizes a capacitor and connects the appliance again [20].

The NREL in [21] presented research focused on plug load control and behavioral change in office buildings. The study consisted of a deployment of advanced power strips (APS) in GSA offices along with two plug load reduction strategies: schedule timer and load sensing. Under the test conditions, APS implementation resulted in an average electricity savings of 21% for laptops, 35% for printers, 7% for monitors, 12% for under-cabinet lights, and 48% for shared equipment (office and kitchen combined). The APS characteristics were four receptacles for plug-in devices, a fuse that trips at 1800 watts (W) and a manual reset button, which allows the user to override the controls that were programmed into the device. The APS used does not have direct internet communication so it has to transfer data through Zigbee to a gateway, which must be within 50 m (164 feet) of the APS.

Park et al. proposed in [22] a Smart Energy Management Systems SEMS based on a smart power strip and motion sensors. The power strip uses ZigBee wireless communication and relays to control sockets, as well as current transformers and an integrated circuit to measure energy consumption in individual plugs. The SEMS can turn on/off loads in two ways, depending on the test room activity based on motion sensors (whether or not people are present) or according to a predetermined time of use. This SEMS does not use an IoT architecture but rather has a computer server that allows the user to set timers and view only the power and energy consumption.

In [8], Spanò et al. proposed an architecture for a smart meter based on the Internet of Things with the intention to be part of the smart grid infrastructure. The scheme presented is focused on the end-user in order to enable smart home applications such as smart plugs. The device is capable of turning on/off electronic devices and also providing some electrical parameters such as active, reactive and apparent power, power factor, and rms current and voltage. The smart plug is based on the energy measurement unit ADE7953, and it uses a shunt resistor as a current sensor, which requires an invasive application to function. This outlet does not have direct internet communication, but it transmits directly to a gateway using ZigBee technology, which is responsible for sending all the information to the cloud.

In the same way, Tsai et al. [23] worked on a residence energy control system based on a wireless smart socket and IoT. Their implementation has three major components including smart socket, home gateway, and energy controller. The smart socket was equipped with a digital power meter which supports between 50 V and 350 V, and current from 10 mA to 15 A. The smart socket itself does not have direct internet communication. It transmits the information via ZigBee to a gateway equipped with 64 MB SDRAM, ZigBee module, 100 Mbps Ethernet inter- face, and USB I/O interface. The energy controller was developed on a server with an Intel i5-2300 2.8 GHz processor, 16 GB RAM, 1 TB hard disk, and a Linux 3.8.13 operating system. With all that hardware, the system provides four control modes, including peak-time control, energy-limit control, automatic control, and user control. In addition, they showed how the proposed scheme could save up to 43.4% of energy for some appliances in one weekday, but there is no electrical parameters’ validation.

Pawar and Vittal K in [2] worked on the design and development of an intelligent energy management system integrated into the IoT framework and addressed to a smart grid environment, which is based on a smart socket module. The electronic circuit they made is big compared to conventional plugs because it is built based on existing electronic modules, such as the Arduino Uno, a relay module for load control and an Xbee for wireless communication. It also has the LEM LV-25P voltage transducer which requires a transformer to be implemented and the LEM LA-55P current transducer. Their system only provides the electrical parameters of rms current and voltage, apparent power, power factor, and energy in watt-hours. As in [8], the smart socket module does not have direct internet communication, so it sends all the collected data by Xbee to a gateway, which can upload the information to a cloud database.

Similarly, in [24], an IoT smart socket for electricity control in a home environment was presented. The system uses two invasive current sensors, two relays to switch on/off up to two loads per device, an AC/DC converter to supply the whole circuit from the line power, and the Wemos D1 Mini development board with a WiFi module to control the complete system and enable internet communication. All the components and connections were enclosed inside a wall socket. However, the system did not include any voltage sensors, so in order to compute the power consumption, a voltage of 220 V rms was assumed. In the web application, the user can monitor current from the smart socket plugged, turning on/off the electricity switch manually and setting a timer for turning on/off the smart socket.

An Internet of Things smart energy meter for monitoring energy usage in a device-level was presented in [25]. Their concept consists of an outlet capable of obtaining rms current and voltage and active power and energy, but it does not have the feature of controlling the load of what is connected to it. Karthick et al. in [26] designed and built an IoT-based smart compact energy metering system to monitor and control energy usage and power quality with demand-side management for a commercial building. In their scheme, there are groups of primary and secondary loads to control and monitor their consumption, but there is not a measurement of energy in individual household devices. The system as a whole has a distributed architecture, which has a central measurement system based on the PZEM-004T (sensor with an invasive application) and different smart switches. Each component uses the ESP8266 to communicate with a Raspberry Pi, which is responsible for calculating some other electrical parameters and sending the information to the cloud.

To conclude with this section, ref. [27] conducted an investigation that provided valuable information for the design and implementation of smart energy management systems. The authors focused on providing a better understanding of user perception and motivations when adopting energy management systems for plug loads in the workplace. With a comprehensive analysis of what they obtained in the research, they proposed seven design implications that could improve the following areas in SEMS: external and internal influence, user appeal, user control, reliability, ease of use, personalized and contextualized information, and data privacy. The same authors, but in [28], worked with strategies to improve the implementation of plugs with load control. Tekler et al. state that real-world applications in this area remain relatively unexplored due to several issues related to deployment viability, energy-saving potentials, and system acceptance. For the above, they presented a novel IoT-based occupancy-driven plug load management system, called “Plug-Mate”, designed to reduce plug load energy consumption and user burden through intelligent plug load automation. The researchers spread 30 smart plugs inside a university office space that recorded users’ real-time plug load power consumption, which was transmitted to a gateway device via Z-wave communication protocol. They proposed and applied different levels of plug load automation, including manual, predefined schedules, and occupancy-driven, all of them implemented from an online user interface. With the above strategies, they achieved an average energy savings of 51.7% among different plug load types evaluated.

### Discussion

As a result of the study of the existing works that address home energy management systems by developing and implementing devices with load control and energy monitoring, it was concluded that there are aspects that need to be improved. For example, those based on IoT architectures that require more than one device for internet connection make it more difficult for users to implement and adapt. In addition, some approaches do not use their own electronic circuits, limiting them to the characteristics provided by smart meter manufacturers or electronic module manufacturers. Additionally, customized circuits make it easier to develop devices that are scalable with standardized electrical systems.

Likewise, how current is measured is a topic to take into account. An important drawback of invasive sensors, such as shunts, is that they are unavoidably electrically connected to the current to be measured and the sense circuit, which means there is no isolation and that the whole circuit is less protected. The above is not the case of current transformers or some Hall effect sensors [29].

Even though the main idea in HEMS is the efficient consumption of energy, there is no reason to only present this electrical parameter. Smart meters with load control that provide more electrical parameters (such as active, reactive, apparent power, power factor, line frequency, etc.) can be used in a wide range of scenarios, including detecting appliance failures, or detecting loads automatically with machine learning algorithms.

Furthermore, how the user interacts with energy management systems is very important for adaptation. Some applications only focus on automatic control and do not provide direct manual control for the user. Some others, however, use manual control only and do not incorporate automatic functions to make their systems more efficient. In addition, many studies do not present how their devices were calibrated and a validation of their measurements, which adds an uncertainty regarding how correct the information they provide is. Moreover, a demonstration of how the proposed system functions is very important, so it is critical to illustrate how the system behaves with real appliances in real situations.

A comparison is presented in Table 1 among existing works related to the application of energy management systems by developing and implementing devices that control load and monitor energy consumption.

## 3. Description and Development of the Smart Meter with Load Control

**Figure 1:**
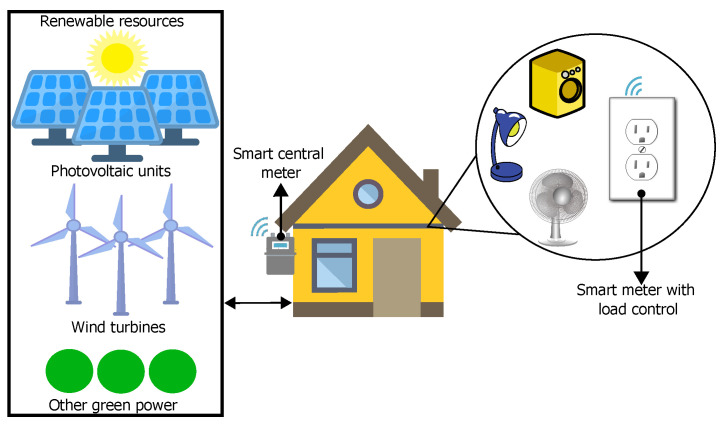
Environment of usage of the smart meter with load control.

In order to have a better understanding of the usefulness of the device proposed in this document, an example of application in an isolated microgrid context can be seen in Figure 1. As mentioned above, with this type of generation and distribution grid, the amount of electricity that homes can use is limited by the availability of renewable sources, and, when power is insufficient, it is necessary to limit it among users. Therefore, the use of a smart central meter placed before the power panel is essential to know how much power is consumed in each dwelling in real time. As a result of the information that is collected for each house and the avoidance of a total power outage in the grid, the smart meter with load control in an outlet format becomes an important tool, since it is capable of sending electrical data (rms voltage and current, active and reactive energy, power factor, and active, reactive and apparent power) through the internet about the electrical equipment connected to it, allowing better and substantiated decision-making. Furthermore, with this device, strategic appliances could be turned on or off remotely, and they could also be categorized in order to schedule and prioritize their usage.

### 3.1. Architecture

**Figure 2:**
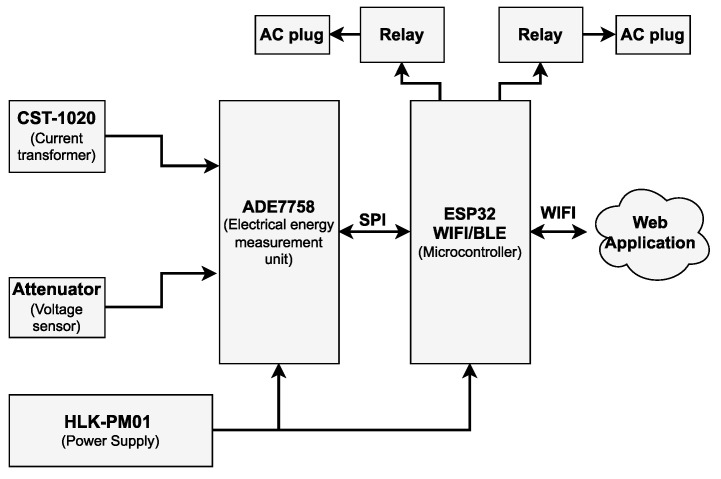
Architecture of the smart meter with load control.

The smart meter and load controller is used here to replace the traditional electrical outlet, measure energy consumption at a device level, and allow the on/off switching of equipment connected to it. In addition, the complete functionality of the system is monitored wirelessly through a web application. In order to achieve the above features, the device’s scheme is built around the ADE7758 as an energy measurement unit, the ESP32 microcontroller, the CST-1020 current transformer, a resistive attenuator for the voltage input, the SRA-05VDC-CL relays, and an integrated power supply HLK-PM01. The architecture used can be seen in the diagram of Figure 2.

#### 3.1.1. Energy Measurement Unit: ADE7758

The ADE7758 is a high accuracy three-phase electrical energy measurement IC that supports the implementation of IEC 60687, IEC 61036, IEC 61268, IEC 62053-21, IEC 62053-22, and IEC 62053-23 standards. It has an SPI serial communication interface and two pulse outputs to interact with external equipment. The ADE7758 incorporates a second order Delta-Sigma type ADC, a digital integrator, reference circuits, a temperature sensor and also the implementation algorithms to determine the active, reactive and apparent power, active and reactive energy, and rms voltage and current calculations, all in a dynamic range of 1000:1. Many three-phase configurations can be used, either for delta or star services of three or four cables, but it can be also implemented for single-phase systems; such is the case in this project.

#### 3.1.2. Microcontroller: ESP32

The ESP32 is a 2.4 GHz Wi-Fi and Bluetooth microcontroller created by Espressif Systems and manufactured by TSMC with 40 nm ultra-low power technology. The product is designed to be robust and reliable in a variety of applications and power scenarios, and to provide optimal RF performance and power consumption. ESP32 is designed for mobile applications, wearable electronics and projects based on the Internet of Things platform. It features all the state-of-the-art characteristics of low-power chips, including fine-grained clock gating, multiple power modes, and dynamic power scaling. In addition, the ESP32 includes a dual core CPU, a 520 KiB SRAME memory, and peripheral interfaces, such as I2C, SPI, I2S, UART, CAN BUS, etc.

#### 3.1.3. Current Sense Input

**Figure 3:**
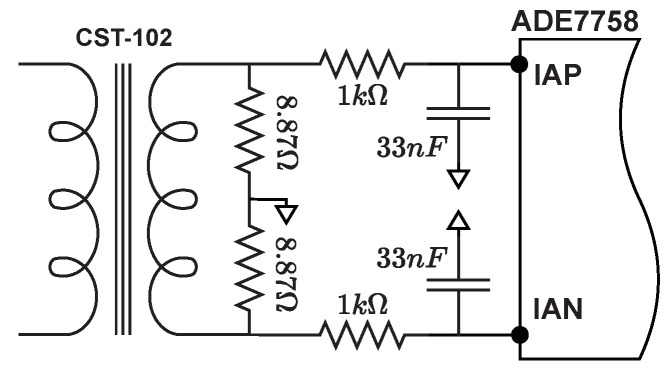
Electronic instrumentation of current input.

The ADE7758 has six analog inputs divided into two sets for current and voltage measurement. The current group consists of three pairs of fully differential voltage inputs: IAP and IAN, IBP and IBN, and ICP and ICN, of which just the first two were used. These fully differential voltage input pairs have a maximum differential signal of ±0.5 V. Due to the above, as well as the size of the traditional electrical outlets and the fact that they typically handle up to 15 A, an insert mount transformer (CST-1020) was used as current sensor. It has a turns ratio of 1000:1 and is capable of handling 20 A. It is also able to operate at 50 Hz as well as 60 Hz. For the electronic instrumentation, shunt-type load resistors were placed at the output of the secondary winding of the transformer to generate a voltage signal that is directed to IAP and IAN. In addition, RC low pass filters with a corner frequency of 4.8 kHz were used on these analog inputs, Figure 3.

#### 3.1.4. Voltage Sense Inputs

**Figure 4:**
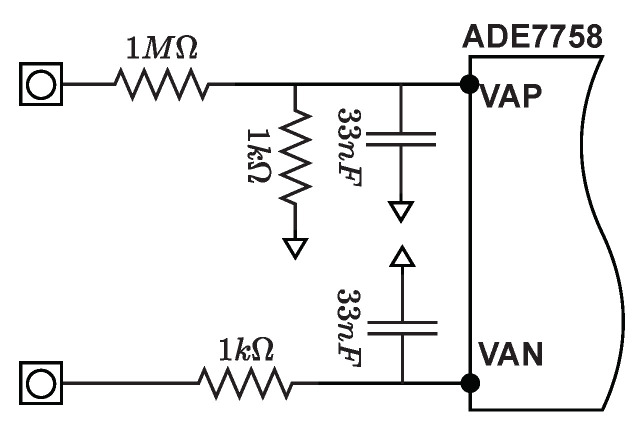
Electronic instrumentation of voltage input which allows a maximum input of 353 V rms.

Figure 4 shows the phase voltage channel signal path on the SMLC circuit. The voltage group has three single-ended voltage inputs: VAP, VBP, and VCP. These single-ended voltage inputs have a maximum input voltage of ±0.5 V with respect to VN. Only VAP and VN were used here. The line voltage is attenuated using a simple resistor divider network before it is presented to the ADE7758. The attenuation network with a ratio of 1000:1 on the voltage channels is designed such that the corner frequency (3 dB frequency) of the network matches that of the RC (anti-aliasing) filters on the current channels inputs.

#### 3.1.5. Load Switcher

In conventional outlets, there are at least two sockets to power different instruments at the same time; this is why the SMLC is designed to switch on/off two plugs individually. This function is achieved by using two electromechanical relays, specifically the SRA-05-VDC-CL. According to its technical specifications, the coil’s nominal voltage is 5VDC, its nominal current is 120 mA, and it can handle loads up to 20 A and switch currents up to 10 A. This relay was chosen because of the good relationship between size and performance.

### 3.2. Printed Circuit Board and Enclosure

**Figure 5:**
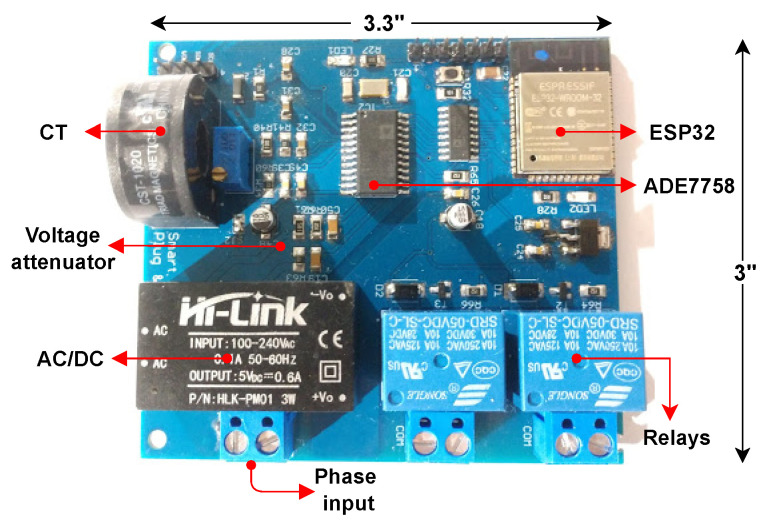
Printed circuit board and components of the SMLC.

**Figure 6:**
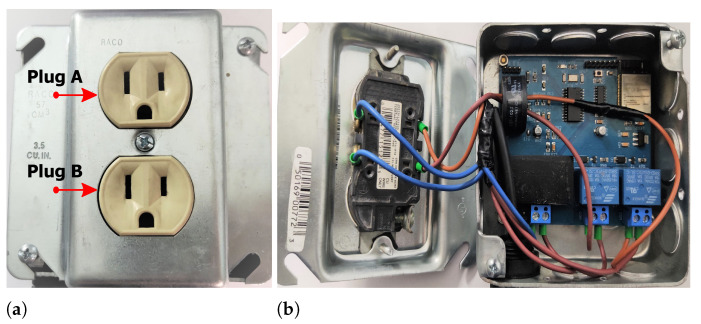
(**a**) Plugs of the SMLC; (**b**) electrical wiring of the SMLC.

All the components of the architecture were taken into account in designing a schematic and a two-layer PCB, which is shown already manufactured with a size of 3.3″ × 3″ and with all the elements soldered in Figure 5. The SMLC aims to replace the traditional domestic electrical outlet so the electronic circuit was placed inside a 4″ × 4″ metal electrical wall box. On the circuit board, neutral and phase were connected to the voltage inputs, the hot wire was also passed through the current transformer, and the relays were wired to a duplex socket, where each plug was labeled as “A” and “B”. Figure 6 is the final prototype of the SMLC.

### 3.3. Calibration

The calibration of the SMLC is a procedure for configuring some registers of the ADE7758 through spi communication, for which the manufacturer supplies a method called line accumulation [30]. In this process, the target is to determine the offset for rms voltage and current, the gain for active, reactive, and apparent power, as well as the phase delay and the offset for active and apparent energy. Part of the calibration process is to measure electrical parameters under different electrical load conditions and compare them with a reference meter, for which the HIOKI PW3360-20 was used, whose characteristics can be found in [31].

#### 3.3.1. Calibration of rms Voltage and Current

Adding an offset to the input signals helps to reduce the noise or previous offset that can appear while a measurement is in process. This can be accomplished by modifying the xIRMSOS (0x36) and xVRMSOS (0x33) registers of the ADE7758. To calculate the value to establish in these registers, the following steps were carried out:

1. Activation of zero crossing detection on the input phase by modifying the LCYCMODE (0x17) register;
2. Modification of the register MASK (0x18) to allow the interrupt pin to be activated with a zero crossing of phases;
3. Set up the calibration system to achieve a test rms current, nominal rms voltage, and minimum rms current and voltage;
4. Average of N samples from the lecture of the registers xIRMS (0x0A) and xVRMS (0x0D) after each interruption caused by the zero crossing detection;
5. Calculation of the offsets with Equations (1) and (2).
  (1) $$\begin{array}{cc} xIRMSOS & =\frac{1}{16384}\times \frac{\left(I_{TEST}^{2}\times IRMS_{IMIN}^{2}\right)-\left(I_{MIN}^{2}\times IRMS_{ITEST}^{2}\right)}{I_{MIN}^{2}-I_{TEST}^{2}}. \end{array}$$
  (2) $$\begin{array}{cc} xVRMSOS & =\frac{1}{64}\times \frac{\left(V_{NOM}\times VRMS_{VMIN}\right)-\left(V_{MIN}\times VRMS_{VNOM}\right)}{V_{MIN}-V_{NOM}}. \end{array}$$
6. Adjustment of the registers *xIRMSOS* (0 × 36) and *xVRMSOS* (0 × 33) with the values calculated.

For the calibration system, a test current $\left(I_{TEST}\right)$ of 10 A rms and a minimum current $\left(I_{MIN}\right)$ of 0.052 A rms were used. In Equation (1), $IRMS_{IMIN}$ corresponds to the value of the register xIRMS (0x0A) when $I_{MIN}$ is measured, as well as $IRMS_{ITEST}$ with $I_{TEST}$. The register gave an average of 5221 with a current of 0.052 A rms and 100 samples, whereas with 10 A rms 1,045,378. With the values of the readings, the calculation of xIRMSOS resulted in 140. On the other hand, xVRMSOS was calculated using a nominal voltage $\left(V_{NOM}\right)$ of 123 V rms and a minimum voltage $\left(V_{MIN}\right)$ of 20 V rms. In Equation (2), $VRMS_{VNOM}$ is the reading of the register xVRMS (0x0D) when measuring $V_{NOM}$, and $VRMS_{VMIN}$ when measuring $V_{MIN}$. This register averaged 565,547 with 100 samples of 123 V rms and 94,088 with 20 V rms; therefore, the outcome of Equation (2) was −40.

#### 3.3.2. Gain Power Calibration

This calibration is primarily used to adjust active, reactive, and apparent power measurements. The ADE7758 accomplishes this by utilizing its three registers: xWG, xVARG, and xVAG (0x2A to 0x32), which can be used to increase or decrease the amplitude of the reading. In order to calculate the mentioned gains, the following steps were taken:

1. Clearing of the xWG, xVARG, and xVAG registers;
2. Selection of phase A, B, or C for a line period measurement with register MMODE (0x14);
3. Set up the ADE7758 for the line accumulation mode by writing to LCYCMODE register;
4. Set the number of half-line cycles for line accumulation by modifying the register LINECYC (0x1C);
5. Modification of the interrupt mask with the register MASK (0x18) in order to enable the interrupt signaling the end of the line cycle accumulation;
6. Set up the calibration system. To obtain the gain for active and apparent power, it is necessary to work with a test current and a nominal voltage with a unity power factor;
7. Reset the interrupt status register by reading RSTATUS (0x1A);
8. Read the energy registers xWATTHR and xVAHR after the interruption of line accumulation has occurred and store the values;
  (a) Calculate the values to be written to *xWG* register according to the following equation:
    (3) $$xWG=\left(\frac{WATTHR_{EXPECTED}}{WATTHR_{MEASURED}}-1\right)\times 2^{12},$$
    before obtaining *xWG*, an expected value in the register of active energy must be determined, which is represented by:
    (4) $$\begin{array}{cc} WATTHR_{EXPECTED} & =\frac{4\times 3200\times I_{TEST}\times V_{NOM}\times cos\left(\theta \right)\times AccumTime\times APCFDEN}{1000\times 3600}, \end{array}$$
    where θ represents the phase angle between the voltage and the current, and AccumTime is the total energy accumulation time inside the ADE7758 according to the number of half-line cycles selected. Tacum can be determined as
    (5) $$AccumTime=\frac{No.\;of\;half\;cycles}{2\times line\;frequency\times No.\;used\;phases},$$
    whereas APCFDEN is
    (6) $$APCFDEN=INT\left(\frac{16000\times \frac{V_{NOM}}{V_{MAX}}\times \frac{I_{TEST}}{I_{MAX}}}{\frac{3200\times I_{TEST}\times V_{NOM}}{1000\times 3600}\times cos\left(\theta \right)}\right).$$
  (b) Calculate the values to be written to the xVAG register using the following equation:
    (7) $$xVAG=\left(\frac{VAHR_{EXPECTED}}{VAHR_{MEASURED}}-1\right)\times 2^{12},$$
    $VAHR_{EXPECTED}$ is the same as $WATTHR_{EXPECTED}$ as long as a unity power factor is being used for the calibration system.
9. Write the outcomes of Equations (3) and (7) into registers xWG and xVAG.
10. Set up the calibration system. To obtain the gain for reactive, it is necessary to work with a test current and a nominal voltage with a power factor of 0.5.
11. Repeat step 7.
12. Read the energy register xVARHR after the interruption of line accumulation has occurred and store the values.
13. Calculate the values to be written to the xVARG register using Equation (8):
  (8) $$xVARG=\left(\frac{VARHR_{EXPECTED}}{VARHR_{MEASURED}}-1\right)\times 2^{12},$$
  where $VARHR_{EXPECTED}$ is
  (9) $$\begin{array}{cc} VARHR_{EXPECTED} & =\frac{4\times 3200\times I_{TEST}\times V_{NOM}\times sin\left(\theta \right)\times AccumTime\times VARCFDEN}{1000\times 3600}, \end{array}$$
  and VARCFDEN is calculated as
  (10) $$VARCFDEN=INT\left(\frac{16000\times \frac{V_{NOM}}{V_{MAX}}\times \frac{I_{TEST}}{I_{MAX}}}{\frac{3200\times I_{TEST}\times V_{NOM}}{1000\times 3600}\times sin\left(\theta \right)}\right).$$
14. Write the outcome of Equation (8) into register xVARG.

The calibration system consisted of a nominal voltage of 123.9 V rms at 60 Hz, a test current of 10 A rms with unity power factor, which means there is no phase shift between the voltage and current signals $\left(\theta =0\right)$, and 128 half cycles for the line accumulation time. Once all the electrical physical parameters had been established, the energy consumption measurements were taken. The results were 12,862 and 12,824 for registers xWATTHR and xVAHR, respectively, while, using Equation (4), the expected value was 11,950 for both registers. Utilizing Equations (3) and (7) and the data previously obtained, xWG and xVAG were calculated, resulting in −279 and 290, respectively. According to [30], the gain adjustment for reactive power requires a power factor of 0, which was not achievable with the available loads; therefore, the lowest possible power factor was used: 0.1190, which means an angle of $83.0617^{\circ }$ between the voltage and the current. In this calibration, the nominal voltage was 125.85 V rms, the test current was 5.06 A rms, and the line accumulation was 128 half cycles. This scenario caused the reactive energy register xVARHR to return 6565, while the expected value was 6143 based on Equation (9). Using the previous data in Equation (8), the gain for active power to write in the register xVARG was —264.

#### 3.3.3. Phase Calibration

The ADE7758 includes a phase calibration register in each current channel xPHCAL (0x3F to 0 x41) to compensate small phase errors caused mainly by current transformers, complex phase errors must be fixed by adjusting the values of the antialiasing filters from Figure 3. Phase calibration consists of adding a time delay that can be in a positive or negative direction. To calculate the degree of phase shift of the signal and the value to be written in the xPHCAL register, the following steps were followed:

1. Repeat steps 1, 2, 3, 4, and 5 of the gain calibration to select the phase to calibrate, set the line accumulation mode, define the number half cycles in the line accumulation, and set the interrupt mask;
2. Set up the calibration system. Two active power measurements are required for this calibration, one with a nominal voltage and a test current with a unity power factor and another with a power factor of 0.5;
3. Reset the interrupt status register by reading RSTATUS (0x1A);
4. Read the active energy register xWATTHR after the interruption of line accumulation has occurred and store the values;
5. Repeat steps 2, 3, and 4 but using a power factor of 0.5 in the calibration system;
6. Calculate the phase error in degrees with the Equation (11):
  (11) $$phaseError\left({}^{\circ}\right)=Arcsin\left(\frac{digitalError}{\sqrt{3}}\right),$$
  to determine $phaseError\left({}^{\circ}\right)$ it is necessary to obtain a digital phase error, which can be computed by:
  (12) $$digitalError=\frac{xWATTHR_{pf=0.5}-\frac{xWATTHR_{pf=1}}{2}}{\frac{xWATTHR_{pf=1}}{2}}.$$
7. Find the value to be written in the register *xPHCAL* with the following equation:
  (13) $$xPHCAL=phaseError\left({}^{\circ}\right)\times \frac{9.6\mu s}{Ph/Lsb/W}\times \frac{\frac{1}{line\;frequency\times 9.6\mu s}}{360},$$
  where
  $$PH/Lsb/W=\left\{\begin{array}{cc} 1.2\mu s & digitalError<0\\ 2.4\mu s & digitalError>0 \end{array}\right..$$
8. Modify the register xPHCAL with the outcome of Equation (13).

For the phase calibration, as mentioned in step 2, two active energy measurements are required. The first one was carried out in a unity power factor with a nominal voltage of 123.6 V rms at 60 Hz and a test current of 7.21 A rms. With the above electrical conditions and 128 half cycles for the line accumulation time, the active energy register xWATTHR showed a value of 8641. In the second measurement a power factor of 0.505, a nominal voltage of 123.4 V rms at 60 Hz, and a current of 7.22 A rms were used, resulting in a value of 4470 in the xWATTHR register. A digital phase error of 0.0346 was obtained substituting the measured values in Equation (12), and this datum was used in Equation (11) to find that the phase had a shift of −1.1447°. For the phase error correction, the value to be written in the xPHCAL register was obtained with Equation (13), resulting in a value of −44.

#### 3.3.4. Power Offset Calibration

This calibration serves to meet exceptional performance within the dynamic measurement range of 1000:1, especially when power consumption levels are very low. The ADE7758 has offset registers for the active and reactive power, xWATTOS (0x39 to 0x3B) and xVAROS (0x3C to 0x3E), whereas the offset in the apparent power measurements is affected by adjusting the rms offset registers. This calibration must be performed with a test current as close as possible to the minimum current within the dynamic range, and a greater number of half cycles for the line accumulation is also required to avoid the effect of quantization errors. In order to calculate the power offsets, the following steps were taken:

1. Repeat steps 1, 2, 3, 4, and 5 of the gain calibration to select the phase to calibrate, set the line accumulation mode, define the number half cycles in the line accumulation, and set the interrupt mask;
2. Set up the calibration system with a nominal voltage and a test current achieving a unity power factor;
3. Reset the interrupt status register by reading RSTATUS (0x1A);
4. Read the active energy register xWATTHR after the interruption of line accumulation has occurred and store the values;
5. Calculate the value to be written in the register xWATTOS with:
  (14) $$xWATTOS=\frac{offsetW\times 4}{AccumTime\times CLKIN}\times 2^{29},$$
  where CLKIN is the oscillator frequency used for the ADE7758, and OffsetW can be obtained as
  (15) $$\begin{array}{cc} offsetW & =\frac{xWATTHR_{I_{MIN}}\times I_{TEST}-\left(xWATTHR_{I_{TEST}}\times \frac{No.\;of\;half\;cycles\;I_{MIN}}{No.\;of\;half\;cycles\;I_{TEST}}\right)\times I_{MIN}}{I_{MIN}-I_{TEST}}. \end{array}$$
6. Modify the value of the register xWATTOS;
7. Modify the calibration system with a nominal voltage and a test current at a power factor of 0;
8. Reset the interrupt status register by reading RSTATUS (0x1A);
9. Read the reactive energy register xVARHR after the interruption of line accumulation has occurred and store the values;
10. Calculate the offset for the reactive energy with Equation (16).
  (16) $$xVAROS=\frac{offsetV\times 4}{AccumTime\times CLKIN}\times 2^{29},$$
  offsetV is equal to
  (17) $$\begin{array}{cc} offsetV & =\frac{xVARHR_{I_{MIN}}\times I_{TEST}-\left(xVARHR_{I_{TEST}}\times \frac{No.\;of\;half\;cycles\;I_{MIN}}{No.\;of\;half\;cycles\;I_{TEST}}\right)\times I_{MIN}}{I_{MIN}-I_{TEST}}. \end{array}$$
11. Write the Outcome of Equation (16) in the Register xVAROS

In order to obtain the value of xWATTOS, the calibration system was set to 123.5 V rms as nominal voltage and 0.0525 A rms as a minimum test current with a unity power factor. For the line accumulation, 4096 half cycles were used, resulting in a reading in the active energy accumulation register of 1991, a value that corresponds to $xWATTHR_{I_{MIN}}$. For $xWATTHR_{I_{TEST}}$, a nominal voltage of 123.4 V rms, a test current of 7.2 A rms, and 128 half cycles for line accumulation were used, resulting in a value of 8826 in the xWATTHR register. Once the $xWATTHR_{I_{MIN}}$ and $xWATTHR_{I_{TEST}}$ readings were obtained, both with different accumulation times and a 10 Mhz clock (CLKIN), the offsetW was calculated with Equation (15) giving an outcome of 63, which was used in Equation (14) for xWATTOS, resulting in 397. In the same way, to calculate reactive power offset, a 123.5 V rms voltage and a minimum test current of 0.06 A rms were used, but now with a power factor as close to 0 as possible, 0.1260 in this case. The line accumulation was performed using 4096 half cycles, resulting in a value of 1831 in the reactive energy register, which was used as $xVARHR_{I_{MIN}}$. For $xVARHR_{I_{TEST}}$, a nominal voltage of 123.3 V rms and a test current of 7.22 A rms with a power factor of 0.1260 were used. These electrical conditions, along with 128 half cycles, resulted in a reading of 7655 for the reactive energy register. As a result of the above measurements, offsetV had a value of 206 according to Equation (17), and, using that in Equation (16), xVAROS had a value of 1396.

### 3.4. IoT Integration of the SMLC

**Figure 7:**
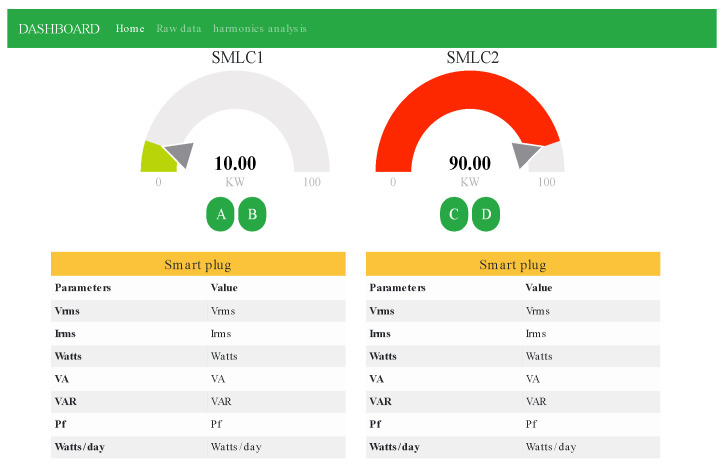
Web application to monitor two smart meters with load control.

One of the novelties of this work is that the SMLC is capable of providing all the electrical parameters previously mentioned in real time through the internet, and it can also receive orders to turn on/off any device that is connected to it. According to [32], there are six levels of IoT implementations and the integration of the SMLC corresponds to the last one because of the following characteristics: (1) it is designed to be an independent end node within a network of multiple SMLCs, the printed electronic circuit developed enables the wide scalability where each SMLC has an internet connection thanks to the ESP32 microcontroller. (2) The information that is sent by the SMLC is stored in a cloud database, specifically MongoDB Atlas. (3) Only electrical variables are calculated by the SMLC; everything else needed is computed in the server. (4) In order to visualize the data, we developed a web app using Node js and Express as a framework, and the PaaS (Platform as a service) Heroku for deploying. The web application can be seen in Figure 7.

**Figure 8:**
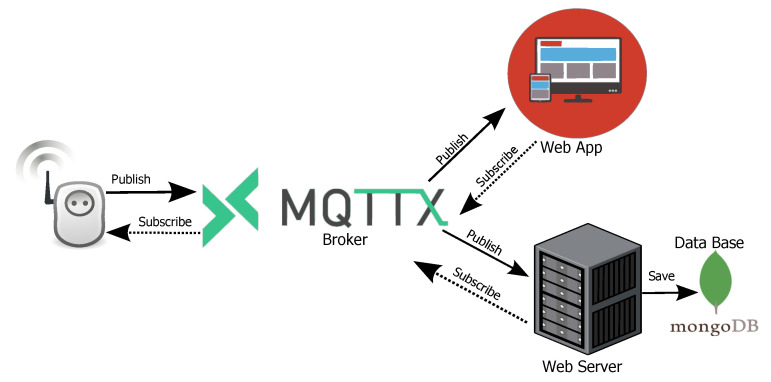
Data flow in the IoT scheme for the SMLC.

It is important to point out that the communication between the SMLC and the server is carried out by the network protocol based on the publish/subscribe method MQTT over TCP/IP sockets, mainly because its publish operation is faster and consumes less energy. On the other hand, to avoid data going through the server and after that to the webapp, we implemented MQTT over websockets to receive data directly from the broker. This technique allows for achieving a latency under 500 ms, from when the measure is taken to it is shown in the cloud interface. An example of how data are transferred can be seen in Figure 8.

## 4. Experiments and Results

### 4.1. Data Validation

**Figure 9:**
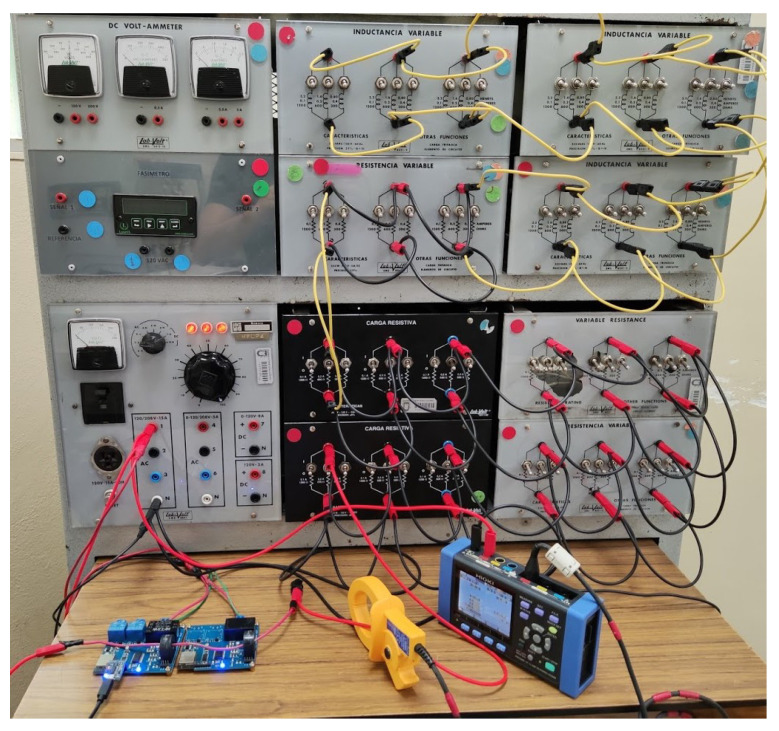
Modular electrical training system used for calibration and validation of the SMLC readings.

In the measurement tests, a modular electrical training system with switchable loads was used to control the physical scenarios of the experiments and thus create different electrical conditions. The modules applied were an AC variable power supply and banks of resistors and inductors. To compare the SMLC readings, the HIOKI PW3360-20 network analyzer was used as a reference measurement instrument. The setup for the validation can be seen in Figure 9.

**Figure 10:**
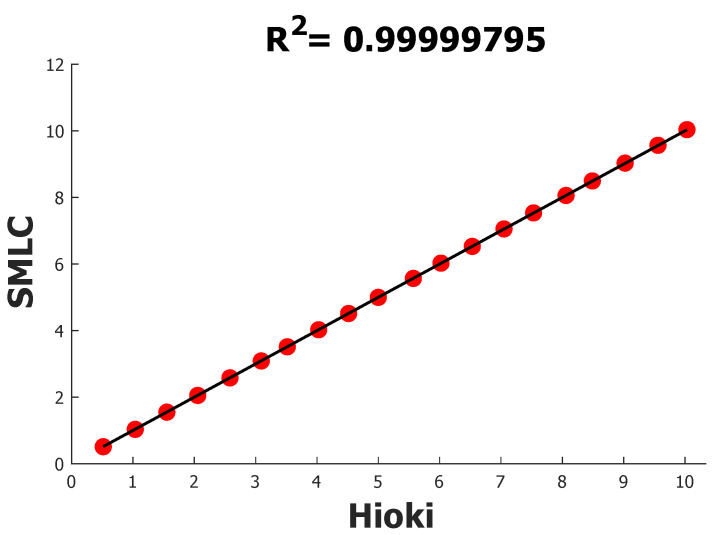
$R^{2}$ of SMLC against HIOKI PW3360-20 using rms current measurements.

In order to achieve better data validation, all the electrical parameters the SMLC can provide were tested twice, once in a unit power factor and once in a 0.5 power factor. The first experiment consisted of making different current values (from 10 A rms to 0.5 A rms) flow through the SMLC using the modules of the power supply (at 127 V rms) and the banks of resistors (pf=1). Under the above conditions, the readings of rms current and active and apparent power were taken. In Table 2, all the outcomes of the SMLC and how they were compared with the HIOKI PW3360-20 measurements can be found. Calculating each error in the readings yields a mean absolute percentage error (MAPE) of 0.1340, and we also carried out a linear regression analysis, Figure 10, to determine the coefficient of determination $R^{2}$ that can be used to weigh how well the SMLC behaves against the reference.

As mentioned in the previous paragraph, in addition to the rms, current other electrical variables were tested, and the $R^{2}$ and the mean absolute percentage errors for all the different parameters are shown in Table 3. For instance, the MAPE for active power was 0.1639 and the $R^{2}$ 0.99999460, whereas the apparent power results were identical to those for active power because, for this experiment, there was no phase shift between current and voltage, which means a unit power factor.

**Figure 11:**
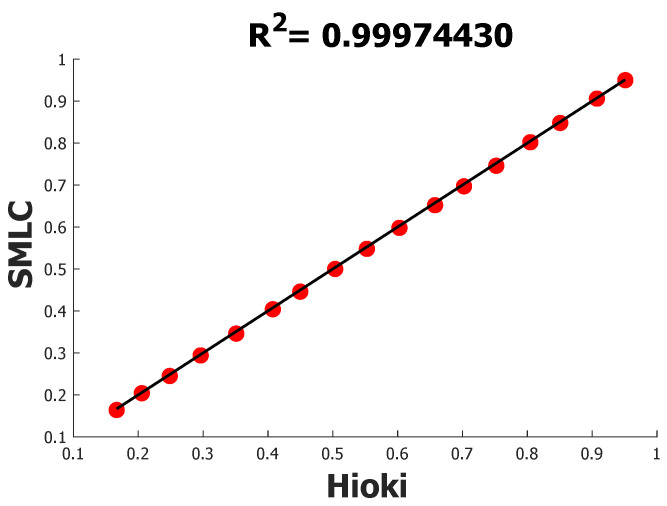
$R^{2}$ of SMLC against HIOKI PW3360-20 using power factors’ measurements.

After finishing the unit power factor trial, distinct power factors were evaluated too, and in order to do so, some banks of inductors were added to the setup to mix them with the resistor loads and thus lag the voltage against the current. Initially, the experiment had a power factor of approximately 0.95, and after that, it gradually decreased in steps of 0.05. The complete readings are in Table 4 along with the error resulting from the comparison with the HIOKI PW3360-20 values, the mean of which was 0.7976%. Moreover, the linear regression analysis is in Figure 11, with an $R^{2}$ of 0.99974430.

Similarly to the last experiment, every time a power factor reading was made, other electrical values were taken, in this case rms current and voltage and active, apparent, and reactive power. To summarize the results, Table 5 contains the MAPE for every parameter according to SMLC and HIOKI PW3360-20 measurements, as well as the corresponding $R^{2}$. In this table, it can be seen that now active and apparent powers have different values; this happens because the power factor is not a unit anymore, which gives a MAPE for active powers of 0.4623% and an $R^{2}$ of 0.99992047, whereas, for the apparent powers, the MAPE was 0.0900% and the $R^{2}$ 0.99994137. It was possible to evaluate the reactive power using this test, which resulted in a MAPE of 0.2443% and $R^{2}$ of 0.99974305.

In order to test how well the SMLC calculates the energy consumption, the source power module (at 126.15 V rms) and the load banks were set, obtaining an rms current of 6.1 A rms and a 0.5 power factor. The SMLC provides this information every second, but to synchronize the kW/h samples with those of the HIOKI PW3360-20, they were compared in steps of one minute. The mean absolute percentage error of the SMLC active energy measurements versus the HIOKI PW3360-20 readings was 0.4242%, and its $R^{2}$ value was 0.99988799, whereas, for the reactive power, it was 0.1095% and 0.999999309, respectively. The last evidence is summarized in Table 6.

**Figure 12:**
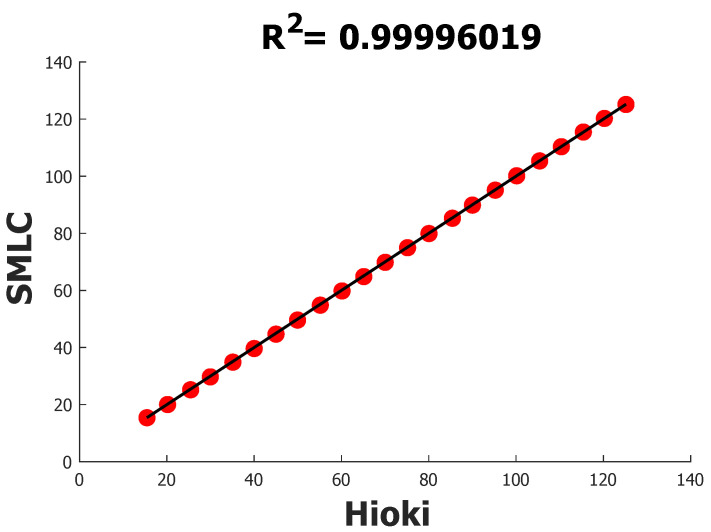
$R^{2}$ of SMLC against HIOKI PW3360-20 using rms voltage readings.

A final experiment was conducted to validate the rms voltage estimation by subjecting the SMLC voltage inputs to values from about 15 V rms to 125 V rms in steps of 5 V rms using the variable part of the power source module. The complete samples are listed in Table 7, along with their respective errors. Considering all the readings, the MAPE resulted in 0.3774% and, according to the linear regression analysis of Figure 12, $R^{2}$ in 0.99996019.

### 4.2. Implementation

**Figure 13:**
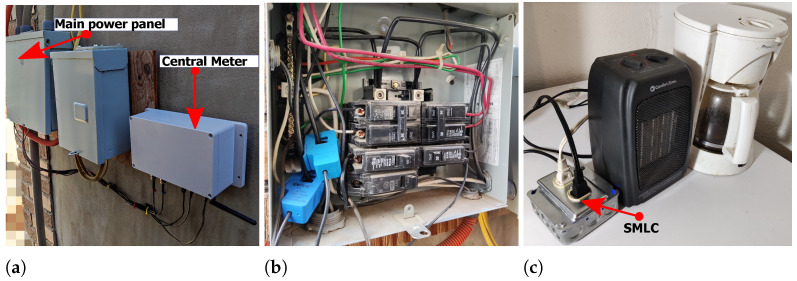
(**a**) Central smart meter next to the main breaker box; (**b**) current transformers of the central smart meter over the two phases of the house; (**c**) coffee maker and electric heater plugged in the SMLC.

To demonstrate its functionality, the SMLC was applied in a real-life operative environment, where it measured electrical parameters and controlled what was connected to it. The setup of the application is shown in Figure 13, where it can be seen how a central smart meter was placed in the main power panel to compare the information provided by the SMLC, which had a coffee maker and an electric heater plugged in; according to the manufacturer, those devices have an active power of 540 W and 720 W, respectively. With the above arrangement, two tests were run, a manual trail where one user turned on and off the loads, and another trail where the loads switched automatically.

**Figure 14:**
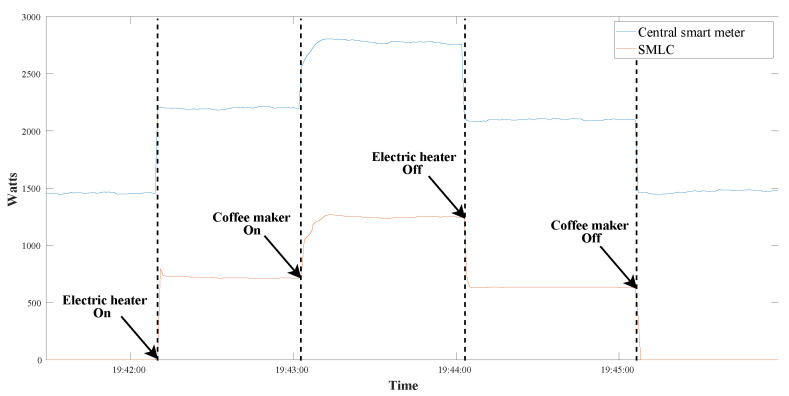
Behavior of the SMLC readings during the manual control of the test loads.

Three states were monitored in the manual test: the first with both appliances off, the second with just the electric heater on, and the third with both devices on at the same time. Figure 14 shows how in the beginning there was no active power in the SMLC while in the whole house there was almost 1500 W. At 19:42:10, the button “A” (from Figure 7) was pressed, causing the electric heater to be turned on, resulting in a reading after a momentary peak of 715 W in the SMLC and a rise to 2184 W in the entire house. After almost one minute, at 19:43:02, the coffee maker was turned on using the button “B” in the web application. The SMLC’s active power went from 715 W to 1245 W after a short transient, which means 530 W for the coffee maker; the central smart meter reported 2776 W. The coffee maker was switched off at 19:43:02, decreasing the active power in the SMLC to 637 W, while the whole house to 2092 W.

**Figure 15:**
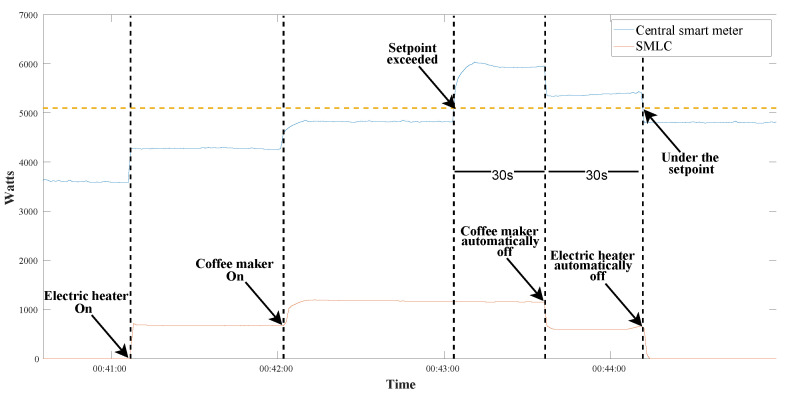
Behavior of the SMLC readings during the automatic control of the test loads.

Another experiment was carried out using the same set up that appeared in Figure 13. This time the loads were switched on/off automatically according to an active power setpoint, which was 5100 W for the entire house. If the power used in the house exceeds 5100 W for 30 s, the SMLC would have to turn off the electric heater or the coffee maker. The first household appliance being switched would be the one connected to plug “B”, and if the total power continues over 5100 W, the next one would be plug “A”. Figure 15 presents the results of the test. At first, the house had a power of 3590 W, then the electric heater and the coffee maker were turned on and the power increased to 4826 W, still under the setpoint. It occurred at 00:43:04 that the power jumped to 5935 W for more than 30 s, which caused the SMLC to turn off the plug “B” (coffee maker), going to 5345 W. After turning off the coffee maker, the total power was still over 5100 W, so 30 s after the first shut-off, the SMLC opened the plug “A”, lowering the power to 4808 W in the house.

## 5. Conclusions

The development of the smart meter with load control for a home energy management system was presented in this paper. The whole practice involved the design and implementation of the electronic instrumentation, the creation of a simple IoT scheme model, a calibration process, the measurement validation, and the demonstration of the system in a space environment.

In contrast to those works that require a gateway to send measurements to the cloud [2,8,21,22,23,26,28], the SMLC electronic proposal based on the ESP32 simplifies the IoT architecture of the entire system because it enables direct internet communication without an extra device. In addition, using integrated circuits specifically designed for calculating electric parameters facilitates future international certifications and assures accurate measurements. Furthermore, the use of a CT as well as the custom-made PCB allows scalability of the prototype in electrical installations, since the circuit board fits in standard 4″ × 4″ metal electrical wall boxes, and, thanks to the CT as a current sensor, a non-intrusive connection can be carried out.

Following the calibration steps for the ADE7758 provided in [30], the SMLC was able to provide readings with a mean absolute percentage error below 0.5% in all its electrical parameters tested with a unit power factor, particularly 0.1340%, 0.1639% and 0.1639% in rms current and active and apparent power. Similarly, but in measurements under no-unit power factor conditions, the MAPE was less than 1%, for example, 0.7976%, 0.0633%, 0.4623%, 0.090%, and 0.2433% in power factor, rms current, and active, apparent, and reactive power, respectively. Active energy consumption exhibited an error of 0.4242% and reactive energy 0.1095%. Readings of rms voltage also showed errors below 0.5%, specifically a MAPE of 0.3774%.

Furthermore, the fact that the SMLC not only provides the active power, but a variety of electrical parameters, including rms current and voltage, reactive and apparent power, and power factor, is an advantage over [20,21,22,24,25,28], as it can be used in future applications. For example, according to Angelis et al. [33], meters that offer the above kind of readings are necessary to implement automatic appliance recognition in HEMS.

Real-time monitoring of electrical energy consumption could not be enough for its efficient use and saving, but it is also essential to facilitate its control. Thanks to the electronic implementation of the SMLC, both functions were possible, and the SMLC includes two electromechanical relays, which demonstrated to be effective switching elements as can be seen in Section 4.2, where they turned on/off different household appliances. In addition, how users interact with energy management systems is crucial to their adaptation. The loads in the SMLC can be switched off/on manually and automatically, unlike [20] that only offer manual control, and [2,21,22] only automatic control.

Future research will focus on how the device proposed in this work could be a useful tool for the areas of smart grids and microgrids, primarily because it allows the opportunity to know exactly how energy is being used in individual appliances, as well as enabling remote control of them—aspects that can help to limit the energy consumption.

## Figures and Tables

**Table 1:** Comparison among existing works that apply energy management systems by developing and implementing devices that control load and monitor energy consumption.

Authors	(1)	(2)	(3)	(4)	(5)	(6)	(7)	(8)	(9)	(10)	(11)	(12)
Kang et al. [20]	No	No	Yes	Yes	No	No	Yes	No	1	No	No	Yes
Metzger et al. [21]	Yes	No	No	Yes	Yes	No	No	yes	4	No	No	Yes
Park et al. [22]	No	No	Yes	Yes	Yes	No	No	Yes	3	No	No	Yes
Spanò et al. [8]	Yes	No	Yes	No	Yes	Yes	Yes	Yes	1	Yes	Yes	Yes
Tsai et al. [23]	Yes	No	Yes	-	Yes	Yes	Yes	Yes	1	No	No	Yes
Pawar et al. [2]	Yes	No	Yes	Yes	Yes	Yes	No	Yes	1	No	No	Yes
Phangbertha et al. [24]	Yes	Yes	No	No	Yes	No	Yes	Yes	2	Yes	No	Yes
Muralidhara et al. [25]	Yes	Yes	Yes	No	Yes	No	Yes	Yes	1	-	Yes	Yes
Karthick et al. [26]	Yes	No	Yes	yes	Yes	Yes	Yes	Yes	-	No	No	Yes
Tekler et al. [28]	Yes	No	No	-	Yes	No	Yes	Yes	1	No	No	No
Us	Yes	Yes	Yes	Yes	Yes	Yes	Yes	Yes	2	Yes	Yes	Yes

^Note:^ (1) Access to Internet; (2) Simple IoT architecture (no gateway needed, on board internet connection); (3) Electronic board design and implementation; (4) Non-invasive current sensor; (5) Energy consumption; (6) Other electrical parameters (V rms, A rms, VA, VAR, pF, etc.); (7) Remote manual load control; (8) Automatic load control; (9) Number of sockets per device; (10) Scalability in standardized electrical installations; (11) Include calibration or validation of measurements; (12) Implementation in a real environment.

**Table 2:** Comparison of rms current samples between HIOKI PW3360 and the SMLC.

HIOKI PW3360	SMLC	%Error
10.031	10.036	0.0498
9.559	9.566	0.0732
9.023	9.03	0.0776
8.489	8.494	0.0589
8.06	8.058	0.0248
7.531	7.535	0.0531
7.049	7.053	0.0567
6.532	6.529	0.0459
6.022	6.026	0.0664
5.571	5.568	0.0539
4.999	4.998	0.0200
4.516	4.512	0.0886
4.028	4.025	0.0745
3.516	3.513	0.0853
3.092	3.088	0.1294
2.582	2.58	0.0775
2.056	2.052	0.1946
1.553	1.55	0.1932
1.038	1.033	0.4817
0.516	0.512	0.7752
**% MAPE**		0.1340

**Table 3:** Resume of all the mean absolute percentage errors and $R^{2}$ of different electrical variables under a unit power factor.

Electrical Parameter	%MAPE	$R^{2}$
Rms current	0.1340	0.99999795
Active power	0.1639	0.99999460
Apparent power	0.1639	0.99999460

**Table 4:** Comparison of power factors samples between HIOKI PW3360 and the SMLC.

HIOKI PW3360	SMLC	%Error
0.9512	0.95	0.1262
0.9074	0.906	0.1543
0.8508	0.848	0.3291
0.8047	0.802	0.3355
0.7522	0.746	0.8242
0.7024	0.697	0.7688
0.6578	0.652	0.8817
0.6028	0.598	0.7963
0.5526	0.548	0.8324
0.5037	0.5	0.7346
0.4499	0.446	0.8669
0.4076	0.404	0.8832
0.3511	0.346	1.4526
0.2963	0.294	0.7762
0.2486	0.245	1.4481
0.2055	0.204	0.7299
0.1667	0.164	1.6197
**% MAPE**		0.7976

**Table 5:** Resume of all the mean absolute percentage errors and $R^{2}$ of different electrical variables under different power factors.

Electrical Parameter	%MAPE	$R^{2}$
Power factor	0.7976	0.99974430
Rms current	0.0633	0.99997885
Active power	0.4623	0.99992047
Apparent power	0.0900	0.99994137
Reactive power	0.2443	0.99974305

**Table 6:** Resume of the mean absolute percentage errors and $R^{2}$ of active and reactive power using 126.15 V rms and 6.1 A rms.

Electrical Parameter	%MAPE	$R^{2}$
Active energy	0.4242	0.99988799
Reactive energy	0.1095	0.99999309

**Table 7:** Comparison of rms voltage samples between HIOKI PW3360 and the SMLC.

HIOKI PW3360	SMLC	%Error
15.44	15.4	0.2591
20.16	20.026	0.6647
25.44	25.19	0.9827
29.95	29.707	0.8114
35.12	34.86	0.7403
40	39.638	0.9050
45	44.672	0.7289
49.94	49.605	0.6708
55.15	54.82	0.5984
60.13	59.812	0.5289
65.11	64.833	0.4254
70.02	69.834	0.2656
75.14	74.946	0.2582
80.05	79.906	0.1799
85.42	85.271	0.1744
90.01	89.874	0.1511
95.24	95.105	0.1417
100.14	100.14	0.0000
105.41	105.349	0.0579
110.38	110.329	0.0462
115.42	115.461	0.0355
120.23	120.241	0.0091
125.18	125.123	0.0455
**% MAPE**		0.3774

## Data Availability

The data presented in this study are available on request from the corresponding author.
